# Genomics as a Clinical Decision Support Tool: Successful Proof of Concept for Improved ASD Outcomes

**DOI:** 10.3390/jpm11070596

**Published:** 2021-06-24

**Authors:** Heather Way, Grant Williams, Sharon Hausman-Cohen, Jordan Reeder

**Affiliations:** 1 The Australian Centre for Genomic Analysis, Brisbane, QLD 4069, Australia; drheatherway@tacga.com.au; 2 IntellxxDNA™, Austin, TX 78731, USA; gwilliams@intellxxdna.com (G.W.); jreeder@intellxxdna.com (J.R.)

**Keywords:** autism spectrum disorder (ASD), genomics, personalized treatment strategy, single nucleotide polymorphisms, clinical decision support tool, ADHD, PANDAS, OCD, anxiety

## Abstract

Considerable evidence is emerging that Autism Spectrum Disorder (ASD) is most often triggered by a range of different genetic variants that interact with environmental factors such as exposures to toxicants and changes to the food supply. Up to 80% of genetic variations that contribute to ASD found to date are neither extremely rare nor classified as pathogenic. Rather, they are less common single nucleotide polymorphisms (SNPs), found in 1–15% or more of the population, that by themselves are not disease-causing. These genomic variants contribute to ASD by interacting with each other, along with nutritional and environmental factors. Examples of pathways affected or triggered include those related to brain inflammation, mitochondrial dysfunction, neuronal connectivity, synapse formation, impaired detoxification, methylation, and neurotransmitter-related effects. This article presents information on four case study patients that are part of a larger ongoing pilot study. A genomic clinical decision support (CDS) tool that specifically focuses on variants and pathways that have been associated with neurodevelopmental disorders was used in this pilot study to help develop a targeted, personalized prevention and intervention strategy for each child. In addition to an individual’s genetic makeup, each patient’s personal history, diet, and environmental factors were considered. The CDS tool also looked at genomic SNPs associated with secondary comorbid ASD conditions including attention deficit hyperactivity disorder (ADHD), obsessive-compulsive disorder (OCD), anxiety, and pediatric autoimmune neuropsychiatric disorder associated with streptococcal infections/pediatric acute-onset neuropsychiatric syndrome (PANDAS/PANS). The interpreted genomics tool helped the treating clinician identify and develop personalized, genomically targeted treatment plans. Utilization of this treatment approach was associated with significant improvements in socialization and verbal skills, academic milestones and intelligence quotient (IQ), and overall increased ability to function in these children, as measured by autism treatment evaluation checklist (ATEC) scores and parent interviews.

## 1. Introduction

Autism spectrum disorders (ASDs) are a group of neurodevelopmental syndromes characterized by deficits in social interaction and communication, as well as repetitive behaviors and restricted interests. ASD rates have increased tremendously over the last few decades from 3 per 1000 children in 1996 to 19 per 1000 children in 2016 [1,2]. While there are forms of ASD caused by pathogenic (disease-causing) genetic mutations, this represents only a small portion of individuals with ASD.

Considerable evidence is emerging that ASD is triggered by the interaction between a variety of single nucleotide polymorphisms (SNPs) and environmental factors such as toxicant exposures, changes to food supplies, and the gut microbiome [3]. In a 2020 study, only 19.7% of individuals with ASD were found to have rare pathogenic variants or copy number variants contributing to or causal of their ASD diagnosis [4]. This indicates that ASD is much closer to what is seen with other chronic illnesses, where a multitude of less common SNPs (found in 1–15% of the population) are likely the main contributors. Additional contributing SNPs may have a much higher population frequency. While individually these SNPs are not disease-causing, they can contribute in an additive manner to the manifestations commonly associated with ASD.

The use of genomics for clinical decision support is a novel approach to medicine that has become feasible only within the last few years. This is in part due to improvements in genetic testing technology as well as advances in the literature regarding the mechanisms of how supplements, nutrients, and other interventions interact with the genome and molecular pathways. This article illustrates how a genomically targeted and personalized medicine approach was successfully used at the Australian Centre for Genomic Analysis (TACGA). While a number of ASD treatment centers across the world incorporate a functional medicine and integrative approach, this is the first time to our knowledge that a specialized neurodevelopmental genomic clinical decision support (CDS) tool has been used systematically to achieve marked improvements in ASD related symptoms. Additionally, as a CDS, the SNPs that were presented and prioritized were actionable. For example, there have been many reports demonstrating the association between elevated tumor necrosis factor alpha (TNFa) and ASD [5], but few studies were identified that connect supplements known to cross the blood–brain barrier and lower TNFa-based inflammation to their usage in response to genetic predisposition to higher TNFa levels.

## 2. Materials and Methods

The four patients presented in this study are a subset of an ongoing pilot study composed of approximately 100 patients and are meant to be illustrative of and give insight into the process used for improving outcomes. Each of the four individuals received treatment for ASD at The Australian Centre for Genomic Analysis (TACGA), which began using genomics in a simplistic manner beginning in 2012. Children who came to TACGA prior to 2018 were evaluated using a basic 54 SNP “health and well-being” panel that included information relating to inflammation, oxidative stress, vitamin D, detoxification and methylation. In 2018, the original version of a neurodevelopmental report (designed to help with non-syndromic ASD) from IntellxxDNA™ (IXXD)—a clinical decision support tool—became available.

IXXD is a more specific CDS tool that offers various versions of its report, including one that focuses on pediatric developmental issues such as ASD, obsessive–compulsive disorder (OCD), pediatric autoimmune neuropsychiatric disorder associated with streptococcal infections (PANDAS), attention deficit hyperactivity disorder (ADHD), and anxiety. This tool was used to analyze DNA specimens from subjects, which consisted of both new and existing TACGA patients. For existing patients, IXXD was implemented as add-on therapy in order to obtain additional improvements in neurodevelopmental outcomes. DNA was collected via buccal cells and analyzed at Rutgers University Cell and DNA Repository (RUCDR) using a customized version of the Affymetrix precision medicine microarray.

Genomic information was presented to the clinician as formatted by IXXD, which was a curated collection of the genomic research. Discussions on gene and SNP function, as well as genomically targeted potential intervention strategies (including nutrients, supplements, and lifestyle modifications) were presented to the ordering provider. In keeping with being a clinical decision support tool, all information was referenced. IXXD reported a particular supplement, food, or nutrient as a potential intervention if it (1) mechanistically addressed both the gene function and SNP impact on the given pathway, and (2) had evidence for improvement of ASD and/or various neurodevelopmental parameters. IXXD nutritional handouts were also incorporated into treatment plans, so that genomics could be addressed with nutrition when possible. A variety of potential intervention options were presented by the CDS, but all treatment decisions were made by the ordering clinician.

The degree of improvements in ASD outcomes were gauged using autism treatment evaluation checklist (ATEC) scores, intelligence quotient (IQ) scores, behavioral improvements, and other parental reporting. In addition to genetic profiling and behavioral observations, TACGA protocol also called for pre- and post-treatment measurement of vitamins, homocysteine, interleukins, and various additional blood markers.

Due to this being a pilot study, there was no specified control group. However, there were individuals evaluated who had previously been optimized with standard TACGA care, who were then given the opportunity to have their genomics evaluated to further improve outcomes. Supplements, nutrients, and dietary modifications were all items that had previously been discussed in the published medical literature and were available over the counter. Thus, consent in this pilot study was obtained via parental discussion.

A detailed discussion on how the CDS tool works is necessary due to this being the first presentation of the IntellxxDNA platform in the ASD literature. For illustration purposes, the Src homology 3 (SH3) and multiple ankyrin repeat domains 3 (SHANK3) variant discussed in the first case study below will be used as an example. Each sentence in quotations below and any accompanying information is linked to references in the live tool. To make the IXXD tool a useful resource for clinicians, genomic reports begin by discussing the gene function and how the variant of interest impacts protein functionality. For example, it would be relayed that SHANK3 “is part of the molecular scaffolding or platform where synapses, especially glutamate receptors of post synaptic nerves, are assembled.” IXXD also provides clinicians with references supporting that the SNP conveys a decrease in SHANK3 expression, along with information linking the associated disorganization of synapses, pervasive developmental disorders, and ASD-like symptoms. Extensive discussions on how each of the SNPs can be modulated are also included. In this example, it is known that SHANK3 is destabilized and broken down by the extracellular signal-regulated kinase 2 (ERK2) protein. Therefore, inhibiting ERK2 with supplements such as curcumin, resveratrol, or a high butyrate diet are presented as potential interventions as they can help raise SHANK3 levels. Additionally, IXXD relays the cofactors that are needed in the molecular pathway, as they can also be modified to improve function. An excerpt from the referenced discussion of SHANK3 modulation in the CDS tool is as follows: “The protein encoded by SHANK3 is regulated by zinc, and zinc deficiency depletes synaptic pools of SHANK3. Melatonin increases SHANK3 protein concentrations. Blue light protection can be beneficial for maintaining proper melatonin levels. ERK2 inhibitors will increase SHANK3 protein indirectly by decreasing the degradation of SHANK3. Butyrate is an ERK2 inhibitor. A ketotic diet is one way to increase beta-hydroxybutyrate levels, but high butyrate foods (see patient dietary handout list), in addition to butyrate supplements, can also be beneficial. Resveratrol is also an ERK2 inhibitor and has data in children with ASD. Physical activity has also been shown to increase SHANK3 protein concentrations in the thalamus and cortex.” This detailed information is given for every SNP in the report and differs in complexity, depending on the nature of the SNP itself. Discussions range from complicated SNPs such as SHANK3 and NAD(P)H quinone dehydrogenase 1 (NQO1), down to simple mechanisms such as the nutritional factor phosphatidylethanolamine N-methyltransferase (PEMT) for the choline pathway.

## 3. Results

### 3.1. CJM Case Study

#### 3.1.1. Medical History and Background

The following is a case study of a male patient who initially made significant gains when following TACGA protocol, but later plateaued. His DNA was reevaluated using the IXXD CDS that targeted specific neurodevelopmental and neurobehavioral pathways.

This case study patient, referred to as CJM to protect his identity, was diagnosed ASD level 3 (highest level, requiring substantial support) at age three and intellectually impaired with an IQ of 54 at age seven. On a gluten free/dairy free diet since age five. First presented to the clinic at age 12 with an ATEC score of 117 (neurotypical ATEC score is about 10 or less). He was classified as non-verbal with some occasional rudimentary language in the form of two or three word strings when it suited him, and was unable to follow multiple instructions. Behavioral issues included self-harm, aggressiveness to peers and family, running away, and bed wetting. Also noted was no desire to socialize, lack of attention (2–3 min), lethargy and very low mental energy (less than 2 min), and significant sensory defensiveness around noise, clothing and stimming. Additionally, he displayed hyperactivity at times, a lack of eye contact, and chronic constipation (permanently on laxatives).

In 2015, he was screened using the initial TACGA protocol. He had SNPs relating to interleukin 1 (IL1a and IL1b), vitamin D receptors (VDR) and detoxification pathways. Alongside dietary changes and a gut healing protocol, the interventions were as follows: fish oil (2 g eicosapentaenoic acid + docosahexaenoic acid), broccoli sprouts (releasing 16 mg sulforaphane), vitamin D (3000 IU), anti-inflammatory probiotics, fermented foods in diet, zinc, and D-ribose-L-cysteine (glutathione precursor).

Over the next 12 months, his family reported considerable improvements in both receptive and expressive language. Behavior significantly improved, stimming reduced, and constipation had resolved. His ATEC had reduced to 71, but he subsequently plateaued. Residual symptoms included lack of attention and focus (15 min), lethargy/became mentally tired very quickly (10–15 min), impaired cognitive abilities, bed wetting (still nightly), and some sensory issues (mostly when tired). Language skills were improved, and he was able to talk in phrases and understand most general words, but he struggled to have meaningful conversations. IXXD’s neurodevelopmental report became available for alpha testing in November 2018, and his family decided to pursue this option shortly after in an attempt to break through the plateau.

#### 3.1.2. Genomic CDS Results and Interpretation

Various genomic pathways, including SNPs reported in the literature to contribute to neurodevelopment and cognitive dysfunction, were discovered and appropriately addressed. CJM was found to be homozygous for a relatively rare variant (c.1304 + 48C > T) in the SHANK3 gene, which is found in less than 4% of the population and is highly associated with increased ASD risk. Deletions and variations within the SHANK3 pathway have been associated with ASD [6], and this particular SNP has been associated with an odds ratio (OR) of 5.5 for ASD and an OR of 12.6 for pervasive developmental disorder [7]. This SNP appears to lead to decreased protein activity. SHANK3 variants (or deletions) causing decreased activity are associated with less ability to form glutamatergic nerve connections during brain development and throughout childhood [7]. Furthermore, SHANK3 contributes to delayed or absent speech, lower muscle tone, and altered social interactions [8]. Interventions targeted towards decreasing the breakdown of this scaffolding protein, as discussed in the genomic CDS, were introduced. Some of these interventions included increasing cofactors, such as zinc, that stabilized the SHANK3 protein [9]. Other interventions related to inhibiting ERK2, which is responsible for breaking down SHANK3, included melatonin [10], resveratrol [11], and using blue light filtering glasses (to block decreases in melatonin levels) [12].

This patient also had multiple SNPs that relate to memory and cognition, including mitochondrial membrane issues that predispose him to more oxidative stress and mitochondrial dysfunction. Additionally, CJM had SNPs that disrupt his natural ability to synthesize phosphatidylcholine, which is an essential nutrient for the synthesis of acetylcholine that is also involved in pathways relating to phospholipid membrane production [13]. He was started on citicoline for this PEMT variant, alongside and a variety of supplements for mitochondrial support that included a combined formulation of ubiquinol (UBQH) + pyrroloquinoline quinone (PQQ) and acetyl-L-carnitine. Additionally, targeted anti-inflammatory interventions were addressed with supplements, and dietary changes to support mitochondrial function were instituted (more coconut oil and mildly ketogenic).

ADHD is a frequent comorbidity to ASD [14]. CJM was homozygous for an ADHD-associated SNP found in approximately 7% of the population. This particular SNP can lead to higher glutamate and dopamine, and lower gamma aminobutyric acid (GABA) via serotonin dysregulation [15]. Variants are known to contribute to inattentive ADHD traits [16], reduced impulse control and increased impulsivity [17], and antisocial personality traits [18]. Additional variants were present in pathways that contribute to attention and focus, language delays, and difficulties with auditory processing. Targeted interventions including L-theanine, magnesium threonate, and magnesium citrate were introduced to address some of these additional variants. Over 600 clinically relevant SNPs were evaluated with the neurodevelopmentally focused genomic CDS. Due to the intended brief nature of this case study report, however, we will not go into each of these pathways in great detail.

#### 3.1.3. Effects of Implemented Interventions on CJM

Patient’s bedwetting stopped completely, stimming ceased, cognition dramatically improved, and he is now fully conversational. These new interventions for the multiple mitochondrial related pathways markedly improved his mitochondrial function and energy to the point that he is now able to play tennis and attend the gym regularly.

The changes in this young man’s life have been astonishing. His ATEC score decreased to 21 and IQ increased to 70. CJM was no longer officially classified as intellectually impaired and was legally, according to Australian guidelines, eligible to attend a mainstream school. The patient became class “captain”, attended the end of year prom, passed his driver’s license exam, and was even able to attain part-time employment, working in a gluten free café. Independence became a reality. CJM is now holding meaningful conversations with family, peers, teachers and employer, who are all thrilled with his progress.

### 3.2. JD1 and JD2 Case Study

#### 3.2.1. Medical History and Background

The next two case study patients, referred to as JD1 and JD2, are interesting in that they involve identical twins who presented to the clinic in 2019 at age six. Both patients were reported by the parents to have severely regressed following an early childhood vaccination. Clinically, the children appeared to have symptoms relating to mitochondrial dysfunction and had difficulties with verbal communication.

Although they were identical twins presenting clinically with ASD, one child had additional symptoms more characteristic of ADHD, while the other clinically suffered from severe anxiety and OCD. Both were prone to recurrent PANDAS/PANS flares and OCD symptoms were present and increased during these infections. Prior to genomic interventions, both patients were taking melatonin and low dose naltrexone. IXXD’s neurodevelopmental report was used to elucidate and address some of the root causes not only of ASD, but also of PANDAS/PANS, attention and focus, and anxiety-related symptoms. A table of symptoms prior to and after treatment is presented below (Table 1).

#### 3.2.2. CDS Results and Interpretation

Genetic analysis identified the presence of many different SNPs that correlated with symptoms shared between the twins. The children had a SNP in the mannose-binding lectin 2 gene (MBL2), which plays a role in the complement pathway, a component of the immune system. The T allele of this MBL2 SNP has been associated with significantly reduced MBL2 levels [19]. This correlates with a lower capacity to recognize foreign invaders (such as Streptococcus infections) and a higher risk for PANDAS (OR = 4.15) [19]. The abnormal immune response from these same SNPs has also been associated with brain autoimmune activity [20], reduced blood–brain barrier function [20], tics, and an increased risk of OCD symptoms [19]. To help address this, a combination of lignite to help tighten the tight junctions of the gut [21], vitamin D [22], probiotics [23], and prebiotics [24] were added to the treatment protocol of JD1 and JD2.

The OCD risk was believed to be exacerbated by the presence of two variants in the solute carrier family 1 member 1 gene (SLC1A1). This solute carrier SNP, particularly in homozygotes, appears to contribute to higher glutamate levels [25] and has been shown to be associated with increased risk of OCD behaviors such as hoarding, ordering and lining things up (OR = 2.01) [26]. Targeted interventions including N-acetylcysteine (NAC) [27], L-theanine [28], vitamin D [29] and vitamin B12 [29] were used to address this pathway. Additional SNPs associated with OCD comorbid with tic disorder and severe bed wetting issues (OR = 2.68) [30] were also discovered in JD1 and JD2. As presented in the genomic CDS, there was overlap in potential interventions between SLC1A1 and the additional SNPs (i.e., some of the same supplements could be used to address both pathways).

The twins were revealed to have some SNPs that are fairly uncommon, as is the case with most TACGA patients presenting with ASD. They were shown to be homozygous for a rare protein kinase SNP found in just 5% of the population. Variants have been associated with increased ASD risk (OR = 1.86) [31], and are involved in pathways relating to cell differentiation, autophagy and survival, and brain development and remodeling [32]. Resveratrol and NAD+ were incorporated into the treatment protocol since both have been shown in studies to help autophagy pathways [33,34].

SNPs contributing to mitochondrial dysfunction were present in JD1 and JD2 and were believed to impact severe fatigue, and may have also contributed to some of their muscle weakness, as evidenced by trouble holding own posture and pencil grip. Genetic analysis revealed that both children had a variant in NQO1 that is associated with an approximate 67% reduction in enzymatic activity [35]. This contributed to mitochondrial dysfunction, oxidative stress, and impaired ability to clear environmental toxins [35]. To combat this SNP’s low NQO1-conveying effects, both patients were started on sulforaphane, which is known to upregulate NQO1 activity [36]. Additionally, NQO1 is needed to convert coenzyme Q10 (CoQ10) to its active form ubiquinol [37]. Therefore, ubiquinol was also used to address this pathway.

Variants relating to vitamins, ADHD, neurotransmitter balance and various other molecular pathways were also present in these patients. For the purposes of brevity, a comprehensive discussion of these additional pathways is not included in this case study report. To address some of these other pathways, the twins’ personalized treatment plan included pycnogenol, ashwagandha, pyridoxal-5-phosphate (P5P) and specific soil-borne probiotics.

#### 3.2.3. Post-Treatment Symptoms and Improvements

Highly significant improvements were seen in both JD1 and JD2. Gains in speech and socialization with family and peers were evident. Improvements in sleep were noted, bed wetting ceased, and increased energy levels were obvious. Fine and gross motor skills were improved; the children gained the ability to dress themselves and developed enough coordination to be able to ride scooters. The PANDAS flares decreased in frequency, and marked reductions were noticed regarding OCD and tics. JD1′s anxiety resolved, and he was no longer a picky eater. Parents relayed that he was “eating everything in front of him”. These improvements led to an increase in his weight, and he was no longer considered malnourished. JD2, on the other hand, showed marked improvements relating to ADHD symptoms. ATEC scores in both children have noticeably improved; JD1 showed a 41% reduction in ATEC scores (from 85 to 50), and JD2 showed a 44% reduction (from 97 to 54). ATEC scores continue to improve with each passing month the twins remain on the protocol. Table 1 shows post-treatment symptoms and improvements.

### 3.3. AD Case Study

#### 3.3.1. Medical History and Background

This final case study patient, referred to as AD, will be briefly touched upon. In this case, the patient’s mother chose to go very slowly with supplements—targeted potential interventions were added one at a time. Thus, even though treatment was initiated late in 2019 when his genomic results were initially received, his regimen is continuing to be optimized at time of publication. However, this case is also important in that the CDS allowed better prioritization of interventions, rather than the usual trial and error approach.

Prior to genomics, patient had an ATEC score of 54. Clinically, this male four-year-old exhibited significant language delays with only five words at the age of three, developed a stutter, very frequent hand flapping, tics, eye rolling, stimming triggered by excitement, seizures, inappropriate socializing, and found it hard to focus or concentrate. Child had many chest infections, adenoid surgery, and over 20 rounds of antibiotics and steroids in the previous 12 months. Patient was not on any supplements when he presented to the clinic.

#### 3.3.2. IXXD Genomic Results and Interpretation

AD had many variants known to be of clinical significance in the pathways discussed in both case study patients above, including those associated with the language center and mitochondrial pathways. In addition to the aforementioned pathways and SNPs, it was discovered that the patient had glutamate receptor SNPs as well as two copies of an alcohol dehydrogenase 5 (ADH5) SNP that has been associated with ASD (OR = 1.54) [38]. ADH5 is a glutathione dependent enzyme that is primarily responsible for removing formaldehyde and is also important for protecting natural lipids from peroxidation [38,39]. Formaldehyde is a natural by-product of white blood cells and myeloperoxidase, and when formaldehyde is not properly removed (as would be the case in individuals homozygous for this SNP) it can build up in the brain and become neurotoxic [40,41]. This child’s high rate of infections likely contributed to high neutrophil/myeloperoxidase (MPO) activation and higher levels of formaldehyde. In addition to supporting glutathione levels, since this enzyme is glutathione dependent [42], a list of foods shown to upregulate ADH5 was given the patient’s mother. This list included foods such as pomegranate, watermelon, and tomatoes [43]. AD’s mother was also informed regarding foods that could exacerbate the negative effects of this genomic pathway. For example, it was recommended to avoid foods artificially sweetened with aspartame, since aspartame is converted to formaldehyde [44]. These types of food and supplement interventions were taken from information listed in the referenced IXXD CDS.

Genomic CDS testing revealed that this child was homozygous for the same NQO1 SNP discussed in the case above (but case above only had one copy). Two copies of this SNP are found in approximately 4% of the population and lead to a significant reduction in enzymatic activity (approximately 97%) [35]. This drastic impairment in NQO1 activity contributes to significant mitochondrial dysfunction, increased oxidative stress, and markedly reduced detoxification [37]. As discussed above, NQO1 variants can contribute to decreased levels of the activated form of CoQ10 [37,45]. Being homozygous for this SNP dramatically impaired his ability to detoxify benzene, solvents, and many other pollutants [46]. High levels of these toxicants have been shown to contribute to increased DNA damage when exposed to various pollutants [47]. Unsurprisingly, the patient had extremely high levels of gasoline additives detected in his GPL Tox screen results, which was addressed as well.

Patient was also found to be homozygous for a well-known haplotype in the brain derived neurotrophic factor (BDNF) gene, which is found in approximately 4% of the population. This growth factor has been shown to be very important for memory and mood [48]. These BDNF SNPs contribute to decreased ability to cleave pro-BDNF to the truncated, mature form of BDNF [49]. While the mature form of BDNF is synaptogenic, the pro-BDNF form induces neuronal apoptosis and is synaptoclastic [49]. Furthermore, higher levels of pro-BNDF levels have been observed in patients with ASD [50]. Regular aerobic exercise was encouraged to help increase the conversion of pro- to mature BDNF [51]. A high butyrate diet and butyrate supplement was also implemented to address this pathway [52].

A personalized, genomically targeted treatment plan was developed. Regimen included moderately high dose UBQH-PQQ, sulforaphane, fish oil, L-theanine, butyrate, magnesium threonate, and a few other supplements. Regular aerobic exercise was also encouraged.

#### 3.3.3. Post-Genomic Testing Improvements

ATEC scores with above interventions improved by 54% (scores decreased from 54 to 25) and continue to improve (as per communication with mother). Regarding symptom improvements, speech and socializing improved very quickly upon reducing inflammation and oxidative stress, working on detox pathways, and addressing gut health. Additionally, and remarkably, after adherence to the personalized list of supplements discussed above for only a few weeks, his seizures stopped. Hyperactive behavior continues to decrease, and tics and stims are improving. Parents continue to notice improvements on a weekly basis and are very happy with the progress to date.

## 4. Discussion

Non-syndromic ASD is clearly due to a multitude of contributing genomic factors that interact with environmental factors. The CDS tool used in this study also looked at genomic SNPs associated with secondary comorbid ASD conditions, given that they are pervasive amongst individuals with ASD. Comorbid conditions investigated by IXXD include ADHD, OCD, anxiety, PANDAS/PANS, gastrointestinal issues, food intolerances and nutrient deficiencies. The genomic and environmental factors, however, significantly vary from person to person. Outcomes trials have shown benefit for methyl-B12 [53], sulforaphane [54], luteolin [55], quercetin [56], melatonin [57], vitamin D [58], omega-3s [59], L-theanine [60] and dozens of other supplements in the treatment of ASD and comorbid conditions. Determining which potential interventions would be the most likely to result in improved ASD outcomes in a particular individual, however, has been a difficult hurdle to clear. Evidence-based genomic clinical decision support tools that focus on variants associated with neurodevelopmental, nutritional, toxicant clearing, and inflammatory pathways can help in prioritization and choice of interventions.

These case studies demonstrate that a well-referenced genomic CDS can be used as a tool to aid in the understanding of some of the gene variants contributing to the patient’s neurodevelopmental disorder. This enables clinicians to address root causes and truly personalize treatment strategies, allowing for the achievement of more robust improvements as well as potentially faster improved outcomes in children with ASD. Initial results from the Australian Centre for Genomic Analysis practice using the IXXD tool, as illustrated by these cases, have been extremely promising. This short case series provides optimism for the role of genomics in improving function and quality of life in children with ASD and neurodevelopmental disorders and suggests that genomics in the form of a CDS can decrease the burden of the trial-and-error method.

The first limitation of this case study report is that only four cases were discussed. It will be important to analyze the collective data (ATEC scores, IQ scores, behavioral observations, etc.) from the complete cohort of approximately 100 patients. In this future analysis it will be important to separate out the results from individuals with access to the IXXD neurodevelopmental genomics CDS tool from the beginning, versus individuals who were previously optimized using the center’s previous treatment methods and then plateaued.

A second limitation of this method of addressing neurodevelopmental disorders is that some patients will respond better than others to genomic CDS tools. Additional research must therefore be conducted. Next steps, which are currently in progress, include being able to reproduce the ability to obtain significant improvements in ATEC or other ASD rating scales in private physicians’ offices in ASD centers across the country. In further research, controlled trials comparing the use of genomics to traditional care in ASD would be beneficial. Another limitation of this method is that it is a relatively new field not taught in residency or fellowships, and thus in order for genomics to be used systematically on a larger scale, clinicians will require dedicated time for study and continuing education. Nonetheless, genomics as a CDS tool can shift the paradigm of care for individuals with non-syndromic ASD and allow for higher functioning and better, quicker outcomes.

An additional limitation to this type of personalized medicine is the treatment cost. Utilization of a tailored, genomically-targeted approach is an investment for the family or whomever else is covering the ASD-related expenses. The cost of the IXXD tool used in this study was $900 per patient. The cost of working intensely with a clinical team that is experienced in functional or integrative medicine, genomic interpretation, and nutrition generally ranges from $2000 to $5000 per year. Currently, insurance coverage for genomic testing is most often limited to specific instances (cancer treatment, pharmacogenomics in some situations, whole genomic sequencing for diagnostic purposes). Therefore, the financial responsibility of this IXXD approach is borne by the families. This cost, however, pales in comparison to the multitude of fees that families of children with ASD incur (financing a caregiver, providing special education, loss of wages of family members, etc.). As illustrated in the cases above, there is potential for a significant financial, long-term benefit when a child can improve overall function, attend schools, and join the workforce rather than being fully reliant on caregivers. Ultimately, as additional studies are published showing the benefit of this precision medicine approach, the potential for this type of CDS targeted treatment to become mainstream and covered by insurance is likely to increase.

## Figures and Tables

**Table 1:** JD1 and JD2 symptoms before and after personalized treatment.

	JD1 Pre-Treatment Symptoms	JD1 Post-Treatment Symptoms	JD2 Pre-Treatment Symptoms	JD2 Post-Treatment Symptoms
Behavior	Severe anxiety OCD Obsessed with details Lining things up Severe constipation Not toilet trained Poor sleep Low energy	Anxiety resolved Not afraid anymore Fully toilet trained, dry at night Sleeping well through the night High energy, jumping on trampoline	Hyperactivity Difficulty concentrating Self-harming Meltdowns and rage Poor sleep, night waking Night wetting Low energy	↑ No longer hyperactive Δ Still some difficulty concentrating ↑ Better moods ↑ Sleeping much improved through the night, rarely night wetting ↑ High energy
Speech	Non-verbal Very low receptive language	↑ Speaking in 3–4-word phrases ↑ Initiating conversation with parents ↑ Receptive language good	Considered non-verbal Some echolalia and echolalic “singing”	− Still considered non-verbal − Some echolalia and echolalic “singing” ↑ Listening to commands
Sensory and Cognitive	Very fussy eater, malnourished Severe anorexia Fear of food Rigid rituals around eating food Stimming Low fine motor skills Low gross motor skills	↑ Eating really well, eats anything in sight ↑ Improved fine motor skills ↑ Vastly improved gross motor skills, dressing himself, riding a scooter	Eats well Low fine motor skills Low gross motor skills	↑ Improved fine motor skills ↑ Vastly improved gross motor skills, riding a scooter
Social	Very shy, “in his shell” No eye contact Not engaging with peers or family	↑ Very friendly ↑ Good eye contact ↑ Engaging with family and peers ↑ Playing with brother	Won’t participate in group activities	↑ Much more social ↑ Participates in group activities ↑ Playing with brother
PANDAS—Regular Flares	OCD Facial tics Choreiform hand movements Very high Streptococcus in bloodwork & stool	Δ Still some flares ↑ Markedly less OCD ↑ Facial tics gone Δ Some choreiform hand movements	OCD Verbal tics and humming Very high Streptococcus in bloodwork & stool	Δ Still some flares ↑ Markedly less OCD Δ Occasional Verbal tics and humming

JD1-case study patient; JD2-case study patient; OCD-obsessive compulsive disorder; PANDAS-pediatric autoimmune neuropsychiatric disorders associated with streptococcal infections. Note: ↑ = Significant symptom improvement after treatment; Δ = slight changes/some improvement noted after treatment; − = no change noted after treatment.

## Data Availability

Relevant genomics and data presented in paper. Full access to genomics is part of an online resource available to ordering clinicians and is not available in downloadable or printable form.
